# Diagnostic Value of Uric Acid/Albumin Ratio and Platelet Indices in Predicting Hypervascularization in the Placenta Accreta Spectrum: A Comparative Retrospective Analysis

**DOI:** 10.3390/jcm15010099

**Published:** 2025-12-23

**Authors:** Neval Çayönü Kahraman, Zeynep Şeyhanlı, Gülşan Karabay, Gizem Aktemur, Nazan Vanlı Tonyalı, Furkan Akın, Ali Turhan Çağlar

**Affiliations:** Perinatology Department, University of Health Sciences Etlik City Hospital, Ankara 06170, Turkey; drzeynepseyhanli@gmail.com (Z.Ş.); drgulsankarabay@gmail.com (G.K.); drgizemkizilbuga@gmail.com (G.A.); nazanvanli@gmail.com (N.V.T.); frkanakin@gmail.com (F.A.); turhan_caglar@yahoo.com (A.T.Ç.)

**Keywords:** placenta accreta, hypervascularization, pathologic, platelet count, uric acid, albumin, biomarkers

## Abstract

**Objective**: This study evaluated the association of the uric acid/albumin ratio (UAR) and platelet indices—mean platelet volume (MPV), platelet distribution width (PDW), and platelet large cell ratio (P-LCR)—in predicting hypervascularization in placenta accreta spectrum (PAS) and compared clinical and perinatal characteristics among PAS, placenta previa, and healthy pregnancies. **Methods**: This retrospective study included 229 pregnant women managed and delivered at a tertiary hospital (PAS, n = 76; previa, n = 77; healthy controls, n = 76) between January 2023 and January 2025. Hypervascularization was staged using the ultrasonographic PAS scoring system: PAS0 (placenta previa without hypervascularization), PAS1 (abnormal placental findings without hypervascularization), PAS2 (uterovesical hypervascularization), and PAS3 (extensive vascularity to the parametrial area). The final diagnosis and severity of PAS were confirmed intraoperatively according to the FIGO clinical classification criteria. Platelet indices and UAR were obtained from preoperative blood tests. **Results**: Compared with placenta previa (PAS0) and control groups, PAS1–3 cases had higher gravidity, parity, previous cesarean history, postpartum hemorrhage, hysterectomy, and transfusion rates (all *p* < 0.001). In the high hypervascularization subgroup (PAS2–3, n = 38), MPV (median 10.3 fL) and PDW (11.6%) were significantly lower than in low/absent hypervascularization cases (PAS0–1) (*p* = 0.001, *p* = 0.001, respectively). UAR showed no significant difference (*p* = 0.891). **Conclusions**: Lower MPV and PDW were associated with hypervascularization in PAS and may serve as non-invasive adjunctive markers for risk stratification. Their predictive performance was modest, and UAR had no diagnostic value, likely due to physiological changes in pregnancy. Further prospective, multicenter research is needed to validate these findings.

## 1. Introduction

Placenta accreta spectrum (PAS) is an obstetric disorder characterized by abnormal adherence of placental villi to the myometrium [1]. Its prevalence has increased in parallel with rising cesarean section rates worldwide [2]. Frequently coexisting with placenta previa, PAS is associated with serious maternal and fetal complications, including massive hemorrhage, hysterectomy, preterm birth, and the need for neonatal intensive care [3]. Pathophysiologically, abnormal placental implantation within a uterine scar disrupts the normal uterine wall architecture, facilitating deep trophoblastic invasion and transformation of large uterine vessels. This process results in marked placental hypervascularization, impaired placental separation, and an increased risk of severe obstetric hemorrhage, contributing to substantial maternal morbidity and mortality [4,5]. Although prenatal diagnosis has been shown to reduce hemorrhagic complications at delivery, accurate antenatal identification of PAS remains challenging due to variability in ultrasound equipment, diagnostic criteria, and operator experience [5,6]. To address these challenges, international initiatives such as the European Working Group on Abnormally Invasive Placenta (EW-AIP) and the Delphi consensus have emphasized standardized Doppler-based ultrasound signs to improve diagnostic consistency and risk stratification in high-risk pregnancies [6].

According to the recent international Delphi consensus on PAS ultrasound terminology, the vascular abnormalities seen in placenta accreta spectrum primarily represent hypervascularization, characterized by increased Doppler flow and prominence of pre-existing subplacental and uterovesical vessels rather than formation of new vessels. This hypervascular pattern is thought to arise from deep trophoblastic invasion into a cesarean scar region, where the disruption of the normal uterine architecture leads to exposure and dilation of large peripheral arteries. As a result, ultrasonographic signs such as subplacental hypervascularity, uterovesical hypervascularity, bridging vessels, and intraplacental tortuous vessels—all strongly supported by Delphi consensus—serve as key indicators of abnormal placental adherence. These Doppler-based vascular findings are therefore essential components of prenatal PAS risk assessment, as they reflect the severity of abnormal placental attachment and correlate with increased intraoperative hemorrhage and surgical complexity [4].

Recent cardiovascular research has highlighted the uric acid/albumin ratio (UAR) as a biomarker associated with inflammatory activity, oxidative stress, endothelial dysfunction, and adverse vascular remodeling in cardiovascular and renal diseases characterized by chronic ischemia and microvascular impairment [7,8,9,10,11]. Elevated uric acid levels and reduced albumin concentrations reflect systemic oxidative and inflammatory responses, making UAR a composite indicator of vascular stress.

Although PAS is primarily characterized by abnormal placental adherence and hypervascularization, localized hypoxia and inflammatory activation at the implantation site may contribute to a degree of oxidative stress; however, whether this localized process produces a measurable systemic effect remains uncertain [12]. However, previous PAS studies have predominantly focused on imaging-based diagnostic criteria, and no biochemical marker has been validated to reflect either the degree of placental hypervascularization or the severity of PAS.

Therefore, whether UAR can meaningfully reflect the hypervascularization observed in PAS remains unknown and warrants investigation.

In addition to systemic biochemical markers, platelet indices—such as mean platelet volume (MPV), platelet distribution width (PDW), and platelet large cell ratio (P-LCR)—have emerged as indicators of platelet activation and vascular response [13,14]. Platelets contribute to vascular remodeling by releasing proangiogenic mediators, including vascular endothelial growth factor (VEGF) and stromal cell-derived factor-1α (SDF-1α) [15,16]. Thus, alterations in these indices may offer indirect insight into uteroplacental vascular changes associated with invasive placentation.

Based on these mechanisms, we hypothesized that PAS-related hypervascularization may significantly influence both UAR and platelet indices and that these parameters could serve as non-invasive markers for predicting the presence and severity of PAS. Therefore, the aim of this study was to compare clinical, perinatal, and laboratory characteristics among PAS, placenta previa, and healthy pregnancies, while assessing the diagnostic utility of UAR and platelet indices—particularly those associated with hypervascularization. Identifying reliable biomarkers may support the development of practical, cost-effective tools for the early diagnosis and risk stratification of PAS.

## 2. Materials and Methods

**Figure 1:**
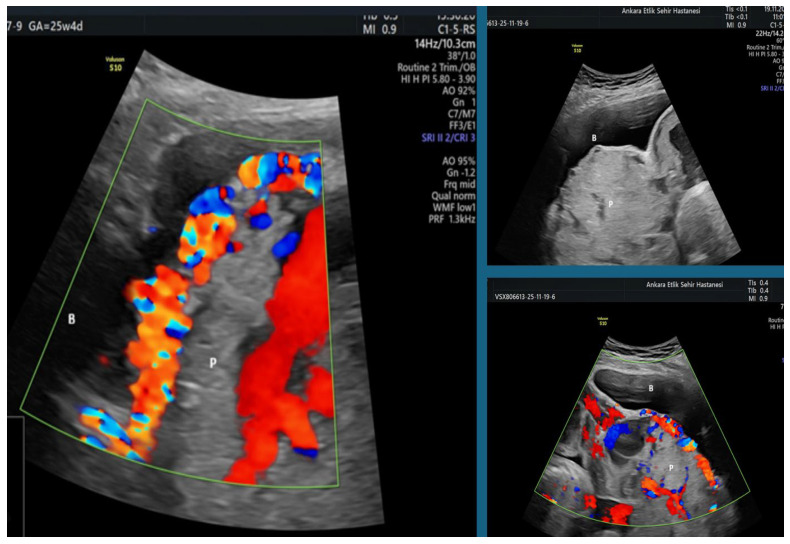
Ultrasound findings of a PAS 2–3 case demonstrating uterovesical hypervascularization, placental bulging toward the bladder, and multiple irregular placental lacunae on a combination of gray-scale and color Doppler imaging (B: Bladder; P: Placenta).

This retrospective study included pregnant women who were followed and delivered at the Perinatology Clinic of Etlik City Hospital, a tertiary referral center, between January 2023 and January 2025. During the study period, a total of 262 pregnant women were consecutively evaluated due to placenta previa and/or suspicion of placenta accreta spectrum (PAS) during perinatology consultations or routine obstetric follow-up. After excluding multiple gestations (n = 19) and major fetal anomalies (n = 14), including three cases with low-lying placenta (two associated with multiple gestations and one with a major fetal anomaly), a total of 229 singleton pregnancies were included in the study. No patient had active vaginal bleeding at the time of blood sampling, and low-lying placentas were otherwise excluded unless they progressed to placenta previa during follow-up. Because PAS and placenta previa are relatively rare conditions, all patients with confirmed PAS and placenta previa—regardless of comorbidities such as gestational diabetes mellitus, hypertension, or preeclampsia—were included in the study. The study population was divided into three groups. The PAS group (G1, n = 76) consisted of patients evaluated during the preoperative ultrasonographic assessment prior to delivery, at which time placental migration is negligible. PAS staging was performed using the Cali PAS 0–3 ultrasonographic scoring system, and all diagnoses of accreta, increta, or percreta were confirmed intraoperatively according to the 2019 FIGO clinical criteria [17,18]. The PAS0/placenta previa group (G2, n = 77) included patients diagnosed with placenta previa, defined as complete or partial coverage of the internal cervical os, whereas low-lying placenta was defined as a placental edge located <2 cm from the os without covering it [19]. Only confirmed placenta previa cases were included, and low-lying placentas were excluded unless they progressed to previa during follow-up. The diagnosis of placenta previa was made during the preoperative ultrasonographic evaluation immediately before delivery, when placental migration had ceased. All patients in this group had placenta previa (PAS0) on ultrasound (absence of standardized PAS signs) and no intraoperative evidence of placental invasion. The healthy control group (G3, n = 76) consisted of low-risk singleton pregnancies with normal placental implantation (fundal or anterior), without placenta previa or suspicion of PAS, and was matched to the other groups for maternal age and body mass index (BMI). All prenatal ultrasound examinations were performed by a single experienced perinatology team, and PAS severity was assessed using the Cali PAS 0–3 scoring system (Table 1, Figure 1). All prenatal ultrasonographic examinations were performed by the same perinatology team, each with at least five years of experience, and PAS severity was evaluated using the Cali PAS 0–3 scoring system (Table 1, Figure 1).

High-resolution ultrasound devices utilized included the Voluson E8 (GE Healthcare GmbH & Co OG, Vienna, Austria). A 5 MHz transducer was utilized for transabdominal assessment, and a 5–9 MHz transvaginal probe was used for transvaginal evaluation. Hematological parameters, including MPV, PDW, and P-LCR, were measured using a Sysmex XN-1000 automated hematology analyzer (Sysmex Corporation, Kobe, Japan). Serum uric acid and albumin levels were analyzed with a Cobas c501 automated biochemistry analyzer (Roche Diagnostics, Mannheim, Germany). Because no universally accepted pregnancy-specific reference intervals exist for MPV, PDW, and P-LCR, these parameters were interpreted using our laboratory’s analyzer-specific reference ranges. All cases were evaluated for depth of invasion, presence of hypervascularization, integrity of the uterine serosa, placental bulge, and the need for hysterectomy. Cases meeting the FIGO 2019 criteria for PAS were assigned to G1, whereas those without evidence of invasion were classified into G2.

For secondary analyses, only patients in the PAS group were further subdivided according to the degree of ultrasonographic hypervascularization: PAS0–1 (low or absent hypervascularization) and PAS2–3 (high hypervascularization) (Figure 1). This classification was used to compare preoperative laboratory parameters and to assess the diagnostic performance of platelet indices through receiver operating characteristic (ROC) curve analyses.

All hematological parameters, including MPV, PDW, P-LCR, and UAR, were obtained preoperatively from routine maternal peripheral venous blood samples analyzed through standard hemogram and biochemistry tests. All blood samples were collected on the same day as the diagnostic ultrasound examination for PAS. Blood sampling was performed as part of routine antenatal evaluation, and fasting status was not standardized because samples were obtained randomly throughout the day. No patients received intravenous fluid therapy before sample collection. None of the patients were experiencing acute bleeding, labor onset, or other acute obstetric complications at the time of sampling.

Platelet indices (MPV, PDW, and P-LCR) were interpreted according to the analyzer-specific reference ranges of our laboratory, as universally accepted pregnancy-specific reference intervals are currently lacking.

The study was approved by the Ethics Committee of Etlik City Hospital (approval no.: AEŞH-BADEK-2025-0308, date: 9 April 2025) and conducted in accordance with the principles of the Declaration of Helsinki. Considering the retrospective nature of the study, the Ethics Committee granted an exemption from the requirement for informed consent.

### Statistical Analysis

Statistical analyses were conducted using IBM SPSS Statistics version 23.0. The normality of continuous variables was assessed using the Kolmogorov–Smirnov test. One-way analysis of variance (ANOVA) was applied for normally distributed variables, while the Kruskal–Wallis and Mann–Whitney U tests were utilized for non-normally distributed variables. Categorical variables were analyzed using the Pearson chi-square test. Post hoc pairwise comparisons with Bonferroni correction were performed to identify the groups responsible for the observed differences. Receiver operating characteristic (ROC) curve analyses were employed to assess the diagnostic performance of platelet indices in predicting hypervascularization. Optimal cutoff values were determined using the Youden index. A *p*-value < 0.05 was considered statistically significant. G*Power software (version 3.1; Franz Faul, University of Kiel, Kiel, Germany) was used to estimate the statistical power of the study (1 − β). Assuming a moderate effect size (d = 0.5), a two-tailed alpha level of 0.05, and equal group sizes for the case group (n = 105) and the control group (n = 105) (total n = 210), the estimated power of the study was 95%.

## 3. Results

The study comprised three groups: PAS1–3 (n = 76, 33.2%), PAS0 (n = 77, 33.6%), and healthy controls (n = 76, 33.2%). No statistically significant differences were observed between the groups regarding maternal age and body mass index (BMI) (*p* = 0.158 and *p* = 0.07, respectively). Significant differences were identified among the groups in terms of gravidity, parity, number of previous cesarean sections, gestational age assessed by ultrasonography, and the presence of comorbidities (*p* < 0.001 for all variables, Table 2).

The rates of conception and delivery were markedly higher in the PAS1–3 group compared with the placenta previa (PAS0) and healthy control groups (*p* < 0.001). The incidence of previous cesarean section was also significantly greater in the PAS1–3 group compared with the other groups (*p* < 0.001). Gestational age determined by ultrasonography was significantly lower in the PAS1–3 and PAS0 groups compared with the healthy group (*p* < 0.001). The prevalence of comorbid diseases was 25% in the PAS1–3 group and 32.5% in the PAS0 group, whereas none of the healthy participants had comorbidities (*p* < 0.001, Table 2).

Birth weight was significantly lower in the PAS1–3 and PAS0 groups than in the healthy group (*p* < 0.001). APGAR scores at one and five minutes were also significantly lower in the PAS1–3 and PAS0 groups compared with the healthy group (*p* < 0.001). The rate of neonatal intensive care unit (NICU) admission was significantly higher in the PAS1–3 group (50%) compared with the PAS0 (29.9%) and healthy (6.6%) groups (*p* < 0.001, Table 3).

In the PAS1–3 group, hysterectomy was performed in 48.7% of cases, whereas all patients in the PAS0 and healthy groups were managed conservatively (*p* < 0.001). Postpartum hemorrhage (PPH) occurred in 25% of PAS1–3 cases and 16.9% of PAS0 cases and was absent among healthy participants (*p* < 0.001, Table 4). The need for blood transfusion was 51.3% in the PAS1–3 group, 16.9% in the PAS0 group, and 1.3% in the healthy group (*p* < 0.001). The length of hospital stay was significantly longer in the PAS group (median 4 days) compared with the previa (3 days) and healthy (2 days) groups (*p* < 0.001).

Post hoc pairwise analyses revealed significant differences between the PAS1–3 group and both the PAS0 and healthy groups in gravidity, parity, previous cesarean history, gestational age, birth weight, APGAR scores, hysterectomy rates, and transfusion requirements. The PAS1–3 group exhibited worse perinatal outcomes across all parameters compared with the other groups.

When comparing patients with high hypervascularization (PAS 2–3, n = 38) and low/absent hypervascularization (PAS 0–1, n = 115), the values of MPV and PDW were significantly lower in the PAS 2–3 group (*p* = 0.001 for both), while P-LCR was not statistically significant (*p* = 0.057). No significant difference was found in the uric acid/albumin ratio (UAR) between the groups (*p* = 0.891, Table 5). Median value comparisons between the groups revealed that the median MPV of 10.3 fL (8.8–13.1) and median PDW of 11.6% (8.9–18.1) in the PAS2–3 group were significantly lower than those in the PAS0–1 group (MPV median 10.8 fL (3–15.1), *p* = 0.001; PDW median 12.7% (3–20.8), *p* = 0.001, Table 5).

**Figure 2:**
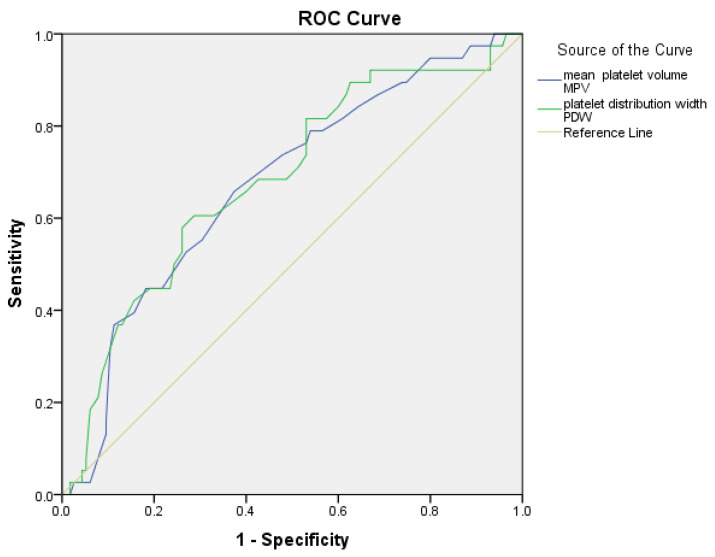
Receiver Operating Characteristic (ROC) curves for platelet indices (MPV, PDW) predicting hypervascularization between the PAS2-3 and PAS0-1 groups.

An ROC curve study was conducted to assess the predictive utility of platelet indicators (MPV and PDW) in diagnosing hypervascularization. Optimal cut-off values for each parameter were determined using the Youden index, and the corresponding sensitivity and specificity values were calculated. The acquired AUC (area under the curve) values are as follows: For MPV, the AUC is 0.675 (95% CI: 0.578–0.771, *p* = 0.001); at a threshold value of 10.55 fL, sensitivity is 66% and specificity is 63%. For PDW, the AUC is 0.683 (95% CI: 0.585–0.782, *p* = 0.001); at a threshold value of 12.35, sensitivity is 66% and specificity is 60% (Figure 2).

The values of MPV and PDW were statistically significant, indicating a significant association with the presence of hypervascularization (*p* < 0.05 for all parameters). In the subgroup analysis, only PAS cases (PAS2–3, n = 38 vs. PAS0–1, n = 115) were evaluated, and the MPV and PDW parameters demonstrated comparable diagnostic performance (Table 6).

## 4. Discussion

This study evaluated the relationship between the uric acid/albumin ratio (UAR), platelet indices (MPV, PDW, P-LCR) and hypervascularization in PAS and found that UAR did not significantly predict hypervascularization severity. In contrast, MPV and PDW demonstrated moderate predictive performance in identifying hypervascularization in PAS. This finding differs from cardiovascular and renal literature, in which elevated UAR levels have been consistently associated with systemic inflammation, oxidative stress, endothelial dysfunction, and a prothrombotic vascular milieu rather than increased blood flow [20]. In these conditions, increased UAR primarily reflects chronic ischemia, impaired microvascular perfusion, metabolic disturbances, and reduced renal uric acid excretion. Such systemic processes substantially influence both serum uric acid and albumin concentrations, leading to an elevated UAR [21,22,23,24,25].

In contrast, pregnancy is characterized by physiological adaptations that influence these biomarkers in the opposite direction. Plasma volume expansion leads to hemodilution and a physiological decline in serum albumin concentration, while increased renal perfusion and glomerular filtration enhance uric acid clearance [26,27]. As a result, UAR values remain inherently lower during a normal pregnancy. Because the participants in our study predominantly consisted of pregnant women without systemic comorbidities known to elevate UAR, the expected biochemical milieu associated with oxidative stress and impaired excretion—frequently observed in cardiovascular populations—was largely absent. Moreover, in PAS, vascular pathology is predominantly localized to the placental implantation site rather than systemic. This physiological and pathophysiological context likely explains the lack of a significant association between UAR and placental hypervascularization in PAS.

Taken together, these findings suggest that UAR, although valuable in chronic ischemic and inflammatory diseases, may have limited prognostic utility in pregnancy-specific conditions such as PAS. The unique metabolic and renal adaptations of pregnancy should therefore be considered when interpreting biomarkers primarily driven by oxidative stress and systemic inflammation.

This study is distinct from previous work by integrating platelet-derived indices into the evaluation of placental hypervascularization in PAS, rather than relying solely on imaging-based markers [28,29,30,31]. Our findings indicate that MPV and PDW exhibit moderate diagnostic performance in differentiating higher-grade hypervascularization (PAS2–3) from lower-grade cases (PAS0–1). Notably, these associations remained statistically significant even after excluding healthy controls, suggesting that platelet indices may reflect the severity of placental invasion among individuals already diagnosed with PAS. The comparable AUC values observed for MPV and PDW support their potential role as adjunctive biomarkers for the extent of placental hypervascularization. Although MPV and PDW were significantly associated with the degree of hypervascularization, their diagnostic performance was only moderate, with AUC values around 0.67 and sensitivity and specificity near 60–66%. These findings indicate that platelet indices alone are insufficient for standalone screening or diagnosis of PAS. Therefore, MPV and PDW should be considered adjunctive markers that may complement, but not replace, established imaging-based assessment. Their clinical utility is likely limited to supporting risk stratification rather than functioning as standalone diagnostic tools. Alternative explanations for the lower MPV and PDW values in the PAS2–3 group should also be considered. Variations in gestational age, subclinical blood loss, and chronic inflammatory conditions may independently influence platelet indices [32]. Although active bleeding and infections were excluded at the time of sampling, these potential confounders cannot be fully eliminated in a retrospective study and should be considered when interpreting the results.

Consistent with prior studies, this study also confirmed that PAS is associated with significant maternal and neonatal morbidity. Higher rates of postpartum hemorrhage, blood transfusion, hysterectomy, and earlier gestational age at delivery—along with lower birth weights and APGAR scores—underscore the severity of this condition [33,34,35]. The strong association between PAS and prior uterine surgery in our cohort aligns with known risk factors and reinforces the need for vigilance in high-risk populations [33]. Taken together, these findings highlight the importance of integrating clinical, laboratory-based, and imaging-based parameters to improve risk stratification and perioperative management in PAS.

This study provides important novel contributions to the existing literature. Unlike previous research that primarily examined platelet indices in preeclampsia or general obstetric populations, our work specifically evaluates their relationship with the degree of hypervascularization in PAS, using a clearly defined and surgically confirmed staging system. By analyzing PAS1–3 and PAS0 groups separately, we demonstrate that lower MPV and PDW values are associated with more advanced PAS stages, supporting a potential adjunctive role for these indices in risk stratification. Furthermore, our findings show that UAR does not correlate with PAS severity, offering additional insight into the limited utility of systemic inflammation–related markers in this context. These results contribute new evidence concerning the hematologic profile of PAS and its potential implications for adjunctive risk stratification.

### Strengths and Limitations

This study’s principal strength is its novel methodological approach, integrating hematological markers—particularly UAR and platelet indices—into the evaluation of hypervascularization in the placenta accreta spectrum (PAS). By comparing clinical, perinatal, and laboratory characteristics across PAS, placenta previa, and healthy pregnancies, the study provides a broader clinical context and contributes meaningful data to obstetric practice. The subgroup analysis restricted to PAS cases further demonstrated that MPV and PDW, obtained through routine and inexpensive laboratory tests, may assist in differentiating the severity of placental invasion. Additionally, the combination of standardized ultrasonographic assessment with intraoperative surgical confirmation strengthens the reliability of the diagnostic classification and outcome interpretation.

Despite these strengths, several limitations should be acknowledged. The retrospective, single-center design may introduce selection bias and limit the generalizability of the findings. The relatively small number of cases with pronounced placental hypervascularization (PAS 2–3) may have reduced statistical power. Although the control group was matched for maternal age and BMI, other relevant clinical variables were not controlled for, allowing the possibility of residual confounding. Additional methodological limitations include the single-time Doppler assessment without interobserver or intraobserver reproducibility testing and the absence of standardized pregnancy-specific reference ranges for platelet indices, which may affect the interpretation of hematological parameters.

Several clinical variables differed between the study groups, including prior cesarean deliveries, comorbid conditions, and gestational age at the time of assessment. These factors may independently influence platelet indices and therefore represent potential confounders in the interpretation of our findings. Because PAS is a relatively rare condition, complete matching of all clinical characteristics across groups was not feasible. As an inherent limitation of the retrospective study design, full control of these variables was not possible and should be considered when interpreting the results.

In addition, laboratory parameters may be influenced by common pregnancy-related hematologic conditions, such as infections, anemia, or gestational thrombocytopenia. These physiological or pathological states can independently affect MPV, PDW, P-LCR, and UAR values and thus represent additional potential confounders. Due to the retrospective nature of the study, these conditions could not be fully controlled and should be acknowledged as a limitation.

The diagnostic performance of platelet indices was modest, with AUC values around 0.67, indicating that these markers are not suitable for standalone clinical decision-making. Moreover, pregnancy-related physiological changes may influence UAR levels and limit its interpretability as a biomarker. Therefore, prospective multicenter studies with standardized protocols and larger sample sizes are needed to further clarify the clinical utility of hematologic markers in PAS.

## 5. Conclusions

In conclusion, placenta accreta spectrum (PAS) remains a critical obstetric condition associated with significant maternal and neonatal morbidity. By integrating clinical features with hematological parameters, this study offers additional insight into the biological characteristics of PAS. Our findings suggest that platelet indices, particularly MPV and PDW, may reflect the degree of hypervascularization and could serve as adjunctive, non-invasive markers to complement established imaging-based diagnostic approaches. Ultimately, accurate risk assessment, timely referral to specialized centers, and multidisciplinary peripartum management are essential for optimizing maternal and fetal outcomes in PAS.

## Figures and Tables

**Table 1:** Ultrasonographic classification of PAS stages (PAS 0–3).

	Definition	Ultrasonographic Features
PAS 0	Placenta previa without hypervascularization	No abnormal placental findings; no evidence of abnormal vascularization on Doppler.
PAS 1	Abnormal placental features without hypervascularization	At least two of: loss of clear zone, presence of placental lacunae, disruption of bladder wall, no hypervascularization.
PAS 2	Uterovesical hypervascularization	PAS1 features plus increased vascularity between the uterus and bladder on Doppler imaging.
PAS 3	Extensive parametrial vascularity	PAS1 or PAS2 features plus marked vascular extension into the parametrial region of the lower uterine segment.

Abbreviations: PAS: Placenta accreta spectrum; BMI: Body mass index.

**Table 2:** Comparison of demographic and clinical characteristics between PAS1–3 (G1), placenta previa (PAS0) (G2), and healthy controls (G3).

	G1 (n = 76)	G2 (n = 77)	G3 (n = 76)	*p*-Value
Maternal age (year) Mean ± SD	32.2 ± 5.1	33.0 ± 6.0	31 ± 4.9	0.158 ^a^
BMI (kg/m^2^) Mean ± SD	29.5 ± 4.2	27.7 ± 5.8	21 ± 4.8	0.07 ^a^
Gravidity Median (min–max)	4 (1–9)	2 (1–9)	2 (1–6)	<0.001 ^b,†^
Parity Median (min–max)	2 (0–7)	1 (0–8)	1 (0–4)	<0.001 ^b,‡^
Number of previous cesarean section Median (min–max)	2 (0–5)	0 (0–3)	0 (0–3)	<0.001 ^b,§^
GW on ultrasound Median (min–max)	32.0 (21.3–39.6)	32.5 (16.2–38.1)	39.0 (27.4–41.4)	<0.001 ^b,¶^
Smoking n (%)	2 (22.6%)	6 (8.0)	1 (1.3)	0.084 ^c^
Additional disease n (%)	19 (25)	25 (32.5)	0 (0)	<0.001 ^c,^*

Abbreviations: BMI: Body mass index, PAS: Placenta accreta spectrum. ^a^: ANOVA test; ^b^: Kruskal–Wallis test; ^c^: Pearson Chi-Square. ^†^: Post hoc Bonferroni corrected Mann–Whitney U test significance between G2 and G1 (*p* < 0.001); G1 and G3 (*p* < 0.001). ^‡^: Post hoc Bonferroni corrected Mann–Whitney U test significance between G1 and G2 (*p* < 0.001); G1 and G3 (*p* < 0.001). ^§^: Post hoc Bonferroni corrected Mann–Whitney U test significance between G1 and G2 (*p* < 0.001); G1 and G3 (*p* < 0.001). ^¶^: Post hoc Bonferroni corrected Mann–Whitney U test significance between G1 and G3 (*p* < 0.001); G2 and G3 (*p* < 0.001). *: Post hoc Bonferroni corrected Chi-square test significance between G1 and G3 (*p* < 0.001); G2 and G3 (*p* < 0.001).

**Table 3:** Comparison of perinatal and neonatal outcomes between PAS1–3 (G1), placenta previa (PAS0) (G2), and healthy controls (G3).

	G1 (n = 76)	G2 (n = 77)	G3 (n = 76)	*p*-Value
GW of delivery (week) Median (min–max)	34.2 (23.4–39.6)	35.6 (24.1–38.3)	39.0 (27.5–42.0)	<0.001 ^a,†^
Birth weight (g) Median (min–max)	2480 (440–3760)	2690 (490–3666)	3205 (1420–4240)	<0.001 ^a,‡^
APGAR 1. min Median (min–max)	8 (2–9)	8 (1–9)	9 (5–9)	<0.001 ^a,§^
APGAR 5. min Median (min–max)	9 (4–10)	9 (4–10)	10 (7–10)	<0.001 ^a,¶^
NICU n (%)	38 (50.0)	23 (29.9)	5 (6.6)	<0.001 ^b,^*

Abbreviations: PAS: Placenta accreta spectrum; GW: Gestational week; APGAR: Appearance, pulse, grimace, activity, respiration; NICU: Neonatal intensive care unit; g: Grams. ^a^: Kruskal–Wallis test; ^b^: Pearson Chi-square. ^†^: Post hoc Bonferroni corrected Mann–Whitney U test significance G1 and G3 (*p* < 0.001); G2 and G3 (*p* < 0.001) originated from. ^‡^: Post hoc Bonferroni corrected Mann–Whitney U test significance G1 and G3 (*p* < 0.001); G2 and G3 (*p* < 0.001) originated from. ^§^: Post hoc Bonferroni corrected Mann–Whitney U test significance G1 and G3 (*p* < 0.001); G2 and G3 (*p* < 0.001) originated from. ^¶^: Post hoc Bonferroni corrected Mann–Whitney U test significance G1 and G3 (*p* < 0.001); G2 and G3 (*p* < 0.001) originated from. *: Post hoc Bonferroni corrected Chi-square test significance G1 and G3 (*p* < 0.001); originated from G2 and G3 (*p* < 0.001).

**Table 4:** Comparison of peripartum and postpartum management and outcomes between PAS1–3 (G1), placenta previa (PAS0) (G2), and healthy controls (G3).

	G1 (n = 76)	G2 (n = 77)	G3 (n = 76)	*p*-Value
Treatment, n (%) Hysterectomy Conservative	37 (48.7) 39 (51.3)	0 (0) 77 (100)	0 (0) 76 (100)	<0.001 ^a,†^
PPH, n (%)	19 (25)	13 (16.9)	0 (0)	<0.001 ^a,‡^
Blood transfusion, n (%)	39 (51.3)	13 (16.9)	1 (1.3)	<0.001 ^a,§^
Length of hospital stay (day) Median (min–max)	4 (2–29)	3 (2–7)	2 (1–4)	<0.001 ^b,¶^

Abbreviations: PAS: Placenta accreta spectrum; PPH: Postpartum hemorrhage. ^a^: Pearson Chi-square; ^b^: Kruskal–Wallis test. ^†^: Post hoc Bonferroni corrected Chi-square test significance between G1 and G2 (*p* < 0.001); G1 and G3 (*p* < 0.001). ^‡^: Post hoc Bonferroni corrected Chi-square test significance between G1 and G3 (*p* < 0.001); G2 and G3 (*p* < 0.001). ^§^: Post hoc Bonferroni corrected Chi-square test significance between G1 and G2 (*p* < 0.001); G1 and G3 (*p* < 0.001); G2 and G3 (*p* < 0.001). ^¶^: Post hoc Bonferroni corrected Mann–Whitney U test significance between G1 and G2 (*p* < 0.001); G1 and G3 (*p* < 0.001); G2 and G3 (*p* < 0.001).

**Table 5:** Comparison between PAS2–3 (high hypervascularization) and PAS0–1 (low or absent hypervascularization) groups.

	PAS 2–3 Groups (n = 38)	PAS 0–1 Groups (n = 115)	*p*-Value
MPV fL Median (min–max)	10.3 (8.8–13.0)	10.7 (3–15.1)	0.001 ^a^
P-LCR % Median (min–max)	27.7 (15.5–46.3)	30.8 (11.1–54.5)	0.057 ^a^
PDW % Median (min–max)	11.6 (8.9–18.10)	12.7 (3–20.8)	0.001 ^a^
UAR Median (min–max)	0.09 (0.05–0.17)	0.10 (0.03–0.26)	0.891 ^a^

Abbreviations: PAS: Placenta accreta spectrum; UAR: Uric acid to albumin ratio; MPV: Mean platelet volume; fL: Femtolitre; P-LCR: Platelet large cell ratio; PDW: Platelet distribution width. ^a^: Mann–Whitney U test.

**Table 6:** Predictive power of laboratory indexes for hypervascularization.

	Cut-Off	Sensitivity (%)	Specificity (%)	AUC	%95 CI	*p*-Value
MPV fL	10.55	66%	63%	0.675	0.578–0.771	0.001
PDW %	12.35	66%	60%	0.683	0.585–0.782	0.001

Abbreviations: MPV: Mean platelet volume; fL: Femtolitre; PDW: Platelet distribution width; AUC: Area under curve; CI: Confidence interval.

## Data Availability

The data that support the findings of this study are available from the corresponding author upon reasonable request but are not publicly available due to ethical and privacy considerations.

## Abbreviations

AUC	Area under the curve
BMI	Body mass index
FIGO	International Federation of Gynecology and Obstetrics
EW-AIP	European Working Group on Abnormally Invasive Placenta
GFR	Glomerular filtration rate
MPV	Mean platelet volume
NICU	Neonatal intensive care unit
PAS	Placenta accreta spectrum
PDW	Platelet distribution width
P-LCR	Platelet large cell ratio
PPH	Postpartum hemorrhage
ROC	Receiver operating characteristic
SDF-1α	Stromal cell-derived factor-1 alpha
UAR	Uric acid/albumin ratio
VEGF	Vascular endothelial growth factor
APGAR	Appearance, pulse, grimace, activity, respiration
